# A Systematic Review of the Impact of Adherence on the Effectiveness of e-Therapies

**DOI:** 10.2196/jmir.1772

**Published:** 2011-08-05

**Authors:** Liesje Donkin, Helen Christensen, Sharon L Naismith, Bruce Neal, Ian B Hickie, Nick Glozier

**Affiliations:** ^1^Brain & Mind Research InstituteThe University of SydneyCamperdownAustralia; ^2^Centre for Mental Health ResearchAustralian National UniversityCanberraAustralia; ^3^The George Institute for Global HealthThe University of SydneySydneyAustralia; ^4^Disciplines of Psychiatry and Sleep MedicineSydney Medical SchoolThe University of SydneySydneyAustralia

**Keywords:** Adherence, persistence, online therapy, e-therapy, systematic review

## Abstract

**Background:**

As the popularity of e-therapies grows, so too has the body of literature supporting their effectiveness. However, these interventions are often plagued by high attrition rates and varying levels of user adherence. Understanding the role of adherence may be crucial to understanding how program usage influences the effectiveness of e-therapy interventions.

**Objective:**

The aim of this study was to systematically review the e-therapy literature to (1) describe the methods used to assess adherence and (2) evaluate the association of adherence with outcome of these interventions.

**Methods:**

A systematic review of e-therapy interventions was conducted across disease states and behavioral targets. Data were collected on adherence measures, outcomes, and analyses exploring the relationship between adherence measures and outcomes.

**Results:**

Of 69 studies that reported an adherence measure, only 33 (48%) examined the relationship between adherence and outcomes. The number of logins was the most commonly reported measure of adherence, followed by the number of modules completed. The heterogeneity of adherence and outcome measures limited analysis. However, logins appeared to be the measure of adherence most consistently related to outcomes in physical health interventions, while module completion was found to be most related to outcomes in psychological health interventions.

**Conclusions:**

There is large variation in the reporting of adherence and the association of adherence with outcomes. A lack of agreement about how best to measure adherence is likely to contribute to the variation in findings. Physical and psychological outcomes seem influenced by different types of adherence. A composite measure encompassing time online, activity completion, and active engagements with the intervention may be the best measure of adherence. Further research is required to establish a consensus for measuring adherence and to understand the role of adherence in influencing outcomes.

## Introduction

The past two decades have seen a shift from traditional face-to-face consultations to technology-driven interventions or e-therapies. Recent reviews have shown that e-therapies are both effective [1-3] and growing in popularity. This is supported by the level of publications relating to e-therapies: Medline and PsycINFO citations in the subject group “online therapy” rose from 12 citations between 1991 and 2000 to 709 citations from 2001 to September 2010.

A potential difficulty in evaluating these programs is adherence (see [4]). Little is known about the degree to which users’ engagement matches the usage pattern for which the websites are designed. Also, little is known about the influence of program adherence on outcomes. Within the medication literature, adherence, “the extent to which a person’s behaviour – taking medication, following a diet and/or executing lifestyle changes, corresponds with agreed recommendations from a health care provider” [5], and persistence, the act of adhering to treatment recommendations for the prescribed duration of time [6], are widely studied. These behavioral variables significantly influence medical [5] and psychotherapy [7-9] outcomes. In pharmaceutical trials, a dose–response curve is often plotted to understand the optimal level of medication to reach a desired response, and adherence is considered highly influential within this. In e-therapy, adherence may be just as important a consideration.

The eHealth equivalent of failing to persist with therapy is treatment dropout. Treatment dropout refers to when a user prematurely stops using the intervention. Some of these users may remain in the trial, completing the trial assessments, while others may choose to leave the trial. Those who choose to leave the trial early are said to have discontinued and are reflected in trial attrition rates. Such attrition can affect the ability of results to be generalized [10,11] and it undermines the statistical power of the trial. Many authors note that dropout rates are high, particularly in open-access trials [12-14] where the intervention is made available to the public with minimal or no cost. Entry into these trials is therefore open, with users being able to join at any time. Despite each user’s choice to engage with the site, only a small proportion of users persist with the trial and associated follow-ups. However, with some programs, these figures may still be comparable to [15], or even lower than [16], the dropout rates found in traditional face-to-face therapies. Little is known about the impact of the degree of persistence on outcome in those who complete trial ratings and outcomes.

While an appreciation of persistence is important in evaluating e-therapy, an understanding of adherence to the program content, such as the completion of program modules or online activities, may be more so. As the field of e-therapy has been growing, so has the interest in potentially modifiable user factors that may influence adherence. Clearly, program content evolves from empirically supported research, but only recently have the exploration and manipulation of program factors become foci. Program usability and feasibility testing is increasingly common. Recent findings have indicated that greater use of computer relational skills, such as the use of empathy and social dialogue in the computer program, leads to increased program usage [17]. Many trials use pilots and usability studies to ensure the program functions as planned [18-22]. Several authors have begun to explore the impact of reminders and trial factors on dropout. Clarke and colleagues’ [23] comparison of the Overcoming Depression on the Internet websites found that participants were more likely to use the program as recommended if they received reminders, and that this increased use was present regardless of the type of reminder (telephone versus postcard) that they received. Alternatively, Christensen et al [24] demonstrated that weekly tracking and reminders reduced attrition in a cognitive behavior therapy e-therapy intervention for depression. Christensen et al [25] also found that shorter cognitive behavior therapy e-therapy interventions were not as effective as longer interventions but that attrition rates were lower, potentially indicating an important trade-off between acceptability and effectiveness. It is thought that the variation in adherence and persistence may be due to the participant’s discretion in using e-therapy [26], rather than therapy that is prescribed, as in a drug trial [13]. Engagement in an e-therapy trial tends to require more physical, cognitive, and time investments by the participant, compared to the relative ease of taking a medication daily. This may explain why e-therapy users are more prone to dropout and nonadherence.

E-therapy trials have an advantage over traditional trials when measuring persistence and adherence, in that more objective measures of intervention usage are readily available to researchers. Objective measures of persistence include metrics such as the number of times the participant accesses the program, and adherence measures include the time spent online, number of completed activities, and patterns of usage. Subjective measures, such as estimated time spent online, reporting on the completion of behaviorally based homework activities, and use of skills, can also be incorporated. Despite the relative ease of capturing these data in online interventions, few studies report these statistics. Merely reporting on adherence and dropout provides limited insight about the impact of adherence on program outcomes or the translation of program behaviors into daily life. Even fewer studies examine the role of adherence on outcomes. The few that do, by publishing only significant results, may result in publication bias.

Recent studies have begun to address this through the exploration of the relationship between program exposure and outcomes [27-30]. Although more data can often be collected in e-therapy trials, there is a need to consider what data are collected, how they are collected, and how adherence is defined. It is likely that the influence of design, application, and logistics of e-therapies on outcomes can become informed in the same way medication dosing affects outcomes. For instance, single daily dosing or polypills have been suggested as methods of improving adherence and outcomes in medication treatments.

To inform this development, we conducted a systematic review of the literature evaluating adherence in e-therapies. Within the context of this review, adherence is defined as the degree to which the user followed the program as it was designed. The two aims were (1) to review the methods used to assess adherence and (2) to evaluate the association between adherence measures and outcome.

## Methods

### Systematic Selection Criteria and Search Strategy

**Figure 1:**
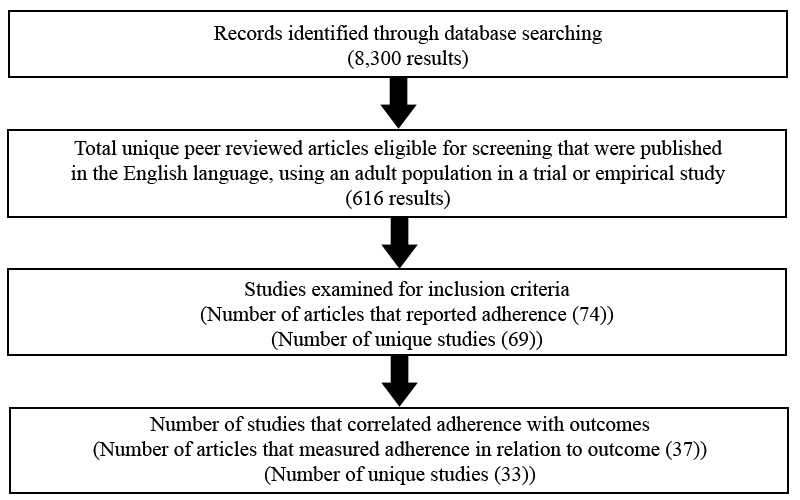
Flow diagram of the process of article selection for systematic review

A systematic database search was used to identify articles relevant to the aims of the review. Articles published up to and including April 2010, as indexed by Medline, PsycINFO, and the Cochrane Central Register of Controlled Trials databases, were included. Broad keyword search terms were used, favoring sensitivity over specificity, given the relative youth of the field. Search terms used were the keywords “Internet” OR “web” combined with “therapy” OR “self-help” OR “intervention.” Following this initial search strategy, we identified 8300 articles (see Figure 1 for a flow diagram of article selection).

The abstracts of the selected articles were reviewed and selected for inclusion by the first author using the following criteria.

#### Inclusion Criteria

An article was suitable for inclusion if it was published in a peer-reviewed journal and the subjects of the intervention were aged 18 years or older. The article also needed to consist of the evaluation of a “self-help,” user-directed online intervention where the Internet was the primary therapeutic delivery modality. In addition, the intervention also required the user to engage with the material on at least 2 occasions in a structured format (therefore, not a website with tools or information, but requiring a progression through the program). When reporting results, the study needed to report 1 or more adherence measures (defined as a measure of program usage by the participant) as well as the outcome of the intervention.

#### Exclusion Criteria

Articles that were not written in the English Language were excluded. Design characteristics that led to exclusion were the participant concurrently receiving a psychotherapeutic intervention in addition to the intervention being studied, or that the program involved synchronous communication as part of the program (eg, online chat, teleconferencing, or personalized therapeutic telephone contact from the therapist during the intervention). These studies were excluded to remove the potentially confounding nature of instantaneous feedback, extrinsic motivation, social desirability, and uncontrolled social contact. Studies with telephone contact were included if the calls were reported to be scripted, described as nontherapeutic in nature, and for the purpose of tracking participants.

Additionally, further studies were excluded because the individual completing the program was not the individual displaying the target behavior (eg, teachers of children with behavioral issues, or parents of children with enuresis), the program was not delivered over the Internet (eg, the program was delivered by CD-ROM via a computer), or the program was delivered at a specific location or time (eg, at a hospital clinic or at a specific time each week), therefore requiring the participant’s presence online at that time or place.

### Coding of Study Characteristics

Key article characteristics were recorded using a data extraction template designed for this review. Key data captured for analysis consisted of variables that would allow the articles to be reviewed and factors thought to be important in adherence, based on a review of the literature.

These factors consisted of study sample size; the study design, including the nature of the control and intervention; the behavior or state that was the target of the intervention; and a description of the intervention, its therapeutic underpinnings, its intervention style, and its requirements from users.

In addition to the study characteristics, adherence data were captured. These consisted of a measure of the degree to which the individual engaged with the website as determined by program logins, engagement in online activities, time spent onsite, and the number of modules completed. Data were extracted for each adherence measure, including the type of measure and data reported relating to this. Any statistical analyses that examined the role of adherence in outcome were also recorded during data extraction.

The data collected on adherence variables were aggregated and evaluated for strength of association with outcome. Due to the methodological challenges associated with Internet trials (eg, high attrition rates), it was difficult to use a widely used rating system. Given these challenges, an alternative rating system was developed, informed by the Scottish Intercollegiate Guidelines Network rating system [31]. Based on this system, the strength of adherence measure rating was determined, using a 5-category scale based on the consistency of findings from this review  (note that only 4 categories are reported here as no measures met the criteria for the 5th category). Details of this system can be found in the footnotes of the corresponding tables.

## Results

We initially found a total of 8300 studies by using the above combination of search terms. Once duplicates were removed, the articles were further limited to those that were published in an English-language, peer-reviewed journal (n = 1095). The remaining articles were systematically reviewed (by LD) to ensure they met the inclusion criteria. This resulted in a total of 74 articles describing 69 individual studies. The heterogeneity of adherence and outcome measures precluded a formal meta-analysis.

Sample size varied significantly between the trials, ranging between 20 and 3176 (median 146), with many of the open-access trials having more participants (Table 1). Structured, discrete-period clinical trials tended to have fewer participants. This may be due to the seemingly more intensive researcher input in the form of feedback provided by emails, monitoring of activity completion, and moderation of activity, requiring greater resourcing for smaller numbers of participants. Larger trials tended to evaluate unstructured websites where participants were able to use the website in whatever manner they pleased, rather than using a structured preplanned program.

Mean study discontinuation rate was approximately 23% of all trial participants (range 0%–83%). Studies of physical and psychological target behaviors had similar attrition rates.

**Table 1:** Descriptive statistics for participant randomization sample size in studies that reported adherence included within systematic review (n = 69)

Descriptive statistic	Total sample size (n)	Physical health sample size (n)	Psychological health sample size (n)
Median	146	190	103
Minimum	20	62	20
Maximum	3176	2523	3176
First quartile	77	91	56
Third quartile	400	958	272
Total number of studies	69	29	40
Total number of participants	34,465	19,147	15,318

### Methods Used to Measure Adherence

Adherence data that were captured varied across studies. This included reporting the number of times the participant accessed or logged into the program, completed modules or activities, visits made to forums, posts made to the forum, and pages viewed and printed, as well as self-reported completion of activities away from the program or offline. Despite the commonality and functionality of being able to capture or measure participant logins across trials, only half (33/69) of the studies presented these adherence data in relation to outcome measures. See table 2 for a breakdown of ways in which adherence was measured in the included studies.

**Table 2:** Methods for measuring adherence to e-therapy as reported by included studies (n = 69)

Measure of adherence	Number of times reported
Logins to program	36
Module completion	31
Time spent online	18
Completion of a predefined activity such or use of an online tool	16
Posts made	9
Pages viewed	5
Replies to emails	6
Forum visits	1
Use of online tools	1
Self-reported completion of offline activities	1
Print requests made	1

Of the 69 studies that reported measuring adherence, approximately half did so by measuring logins and/or completion of modules. Only a quarter of the 69 included studies reported 1 or more of the other potential measures of adherence. The reporting of module completion was more common in studies where the target behavior was psychological health or well-being (25/40, 63%) rather than physical health (6/29, 21%) (n = 69, χ^2^_1_ = 11.9, *P* <.001). Conversely, login reporting was more common in studies where the target behavior was related to physical health (23/29, 79%) rather than psychological health (13/40, 33%) (n = 69, χ^2^_1_ = 14.8, *P* ≤*.*001).

### Effect of Adherence on Outcomes

Of the 69 studies included in the review, 33 (48%) analyzed the impact of 1 or more measure of adherence on outcome variables. Complete results of the review can be found in Multimedia Appendix 1.

#### Logins

A total of 9 studies correlated logins as a measure of adherence to outcomes. Using the previously described rating system, logins were found to be positively associated with outcome in studies targeting food and vegetable consumption [32], physical activity [33-37], and weight management [38-40]. Number of logins was not found to be related to outcomes in studies targeting depression [23,41].

#### Activities Completed

In 6 studies, activities completed were correlated with outcomes. The number of self-reported activities completed (eg, completing a diary, engaging in online tests, or making forum posts) was found to correlate with outcomes in interventions that targeted cigarette smoking [42], weight management [39,40], and body dissatisfaction [43,44]. In studies targeting physical activity [33,34] or depression [45], the number of activities completed was not found to correlate with outcomes.

#### Modules Completed

Completed modules were the most commonly reported measure of adherence, and they correlated with outcomes in 16 studies. The number of modules completed was found to correlate with outcomes in interventions that targeted cigarette smoking [42,46,47], depression [45,48-51], and anxiety disorders [52-57].

#### Time Spent Online

The relationship between time spent online and outcomes was correlated in 4 studies. Time spent online was not correlated to outcomes in studies that targeted depression [58] and anxiety [59], but was correlated to outcomes in studies of infertility related distress [60] and body dissatisfaction [61].

#### Pages Opened

The number of pages opened was explored in 3 studies. The number of pages opened was negatively correlated to outcome in 1 study targeting depression [58] but positively related to outcomes in 2 studies targeting body dissatisfaction [62,63].

#### Website Exposure

The relationship between website exposure and outcomes was explored in 3 studies. Website exposure and program usage was positively correlated with outcomes in interventions targeting smokeless tobacco [64], depression [65], and physical activity [66].

Table 3 summarizes adherence measures by intervention target, reported by target behavior. (For a more in-depth table, please see Multimedia Appendix 1.)

**Table 3:** Summary of the strength of aggregated adherence measure data by target behaviora

		Strength of adherence–outcome association (number of studies)					
Target behavior		Logins	Activities completed	Modules completed	Time spent online	Pages opened	Website exposure
**Physical health**							
	Fruit and vegetable consumption	+^b^ (n = 1)					
	Physical activity	+^b^ (n = 3)	0^c^ (n = 1)				+^b^ (n = 1)
	Weight management	++^d^ (n = 2)	++ ^d^ (n = 3)				
	Smoking		+^b^ (n = 1)	+^b^ (n = 4)			
	Smokeless tobacco						+^b^ (n = 1)
**Psychological health**							
	Depression	0^c^ (n = 2)	0^c^ (n = 1)	+^b^ (n = 7)	–^e^ (n = 1)	–^e^ (n = 1)	+^b^ (n = 1)
	Anxiety			+^d^ (n = 6)	0^c^ (n = 1)		
	Body dissatisfaction		+^b^ (n = 2)		+^b^ (n = 1)	+^b^ (n = 2)	
	Fertility-related distress				+^b^ (n = 1)		

^a^ The number of studies that were aggregated to form the strength rating in the review is indicated in parentheses following the rating indicator (+ = positive; – = negative; 0 = null). (For a complete breakdown of the studies that contributed to the aggregate results see Multimedia Appendix 1.)

^b^ 1 study or mixed evidence with predominantly positive relationships found between adherence measures and outcome.

^c^ No relationship found between adherence and outcome measures.

^d^ At least 2 studies finding a positive correlation between increased adherence and outcome measures.

^e^ 1 study or mixed evidence with predominantly negative relationships found between adherence measures and outcome.

## Discussion

Understanding adherence to e-therapies is important in understanding how these therapies may benefit individuals who need intervention. The impact of adherence on outcome appears to vary. The review demonstrates that however it is measured, adherence is associated positively when reported with intervention outcomes targeting physical health. However, in the most commonly targeted outcome of e-therapy, depression, the number of logins, self-reported activities, time online, and pages opened showed no such association. Only 2 measures of adherence—the degree of completion of the modules within the program and a summative “website exposure” outcome—were associated with better depression outcomes.

The association between the number of logins and outcomes in e-therapies targeting physical health is similar to the positive relationship observed between session attendance and outcome in physical rehabilitation [67-70] and to the dose–response curve seen in medication therapy. This association may largely be due to the number of logins being representative of the participant’s willingness to use the program through their return to the website. The number of logins may be more indicative of program usage than are self-reported activities completed or forums posted. This is particularly the case in programs where there is structured program use (therefore needing completion of an activity or module prior to moving to another) but no time restrictions placed on progression. In this style of program, a participant may complete several modules or activities during a single login. Therefore, the benefits received in completing a module, processing it over a time period, and using its skills before building on this with the next module may be lost.

For participants involved in trials targeting weight changes as an outcome [38-40], all measures of adherence were correlated with outcomes. Therefore, more adherent participants had a higher level of weight loss. This is consistent with the current literature, which shows that the more closely people follow dietary plans, the better their outcomes [71]. In the case of physical outcomes, one interpretation of the results could be that adherence is a marker of an unmeasured factor such as personality or self-efficacy that predisposes participants to a better outcome. A more extreme interpretations is that the content of the intervention is irrelevant; it is the application of the participant to “something” that produces the outcomes.

The most common use of e-therapy is to intervene in depression and anxiety. However, measures of logins, self-reported activity completions, and time online were not associated with outcome—only evidence of actual module completion either on its own or in the composite “program exposure” measure appeared to be related to outcome. This suggests a more nuanced interpretation, in that a participant’s interaction with the module content leads to change, rather than improvement merely reflecting a greater propensity to adhere. This reflects the face-to-face literature, in that the structured psychotherapies, with module (task) completion and review, are generally more effective than supportive psychotherapy [72-74]. The lack of association of many adherence measures with outcome might be explained by lack of power in these studies. This is particularly supported by more consistent findings in physical health interventions, which had a median sample size nearly twice that of psychological interventions. However, it is of note that many psychological health studies did have large sample sizes and, for some adherence measures, the results were not null, but negative.

The definition of adherence and its consequent analysis also varied considerably across studies. Several studies categorized participants into adherent or not [49,54,57,75], or usage categories such as low, medium, and high [60], while others used continuous variables [23,24,33-36,38-41,43-45,50-52,55,56,58,59,61,76] or a combination of these [42,46,53,64-66,77]. Such variation is likely to produce mixed findings. It is also worth noting that several studies reported collecting multiple measures of adherence but reported on the outcome of only a few selected items. In many, it is unclear why some measures of adherence were reported over others, though this may presumably reflect a reporting bias and may therefore inaccurately portray the role of adherence. It is possible that unreported relationships may be nonsignificant, but may also have been useful in understanding the important components of program engagement and may build understanding of the user-engagement factors of e-therapy.

The variation in measurement of adherence in online interventions makes it difficult to accurately determine the impact of adherence on outcome. Despite having several seemingly objective measures such as number of logins, time spent online, and activities completed, there are still difficulties in determining the “dose” a participant receives. Specifically, time spent online is likely to be influenced by factors such as processing speed, cognitive ability, reading aptitude, and familiarly with using computers, several of which are likely to be influenced by psychological health. Likewise, a person who is unfamiliar with using a computer may write out their activities, rather than engage in the functionality presented in the online environment. This needs to be explored further by studies that randomly assign participants to receive different doses of programs, such as that by Christensen et al [25], which explored exposure to modules against change in outcome measures. Future interventions that intend to measure the impact of adherence on outcomes would benefit from clearly defining their adherence variables and exploring all relationships between the potential adherence variables and outcomes. In addition to this, measures of inactivity need to be included where temporal measures are used. Therefore, when a measure of adherence is the amount of time spent online, there needs to be a time-out function of need to engage readily with the program, to show that it is being used. This will allow the exclusion of individuals who have left their computer on and who are no longer engaging with the program material.

From this review, the mechanism of how adherence influences Internet treatment outcomes is unclear. Medical studies have shown that adherence is associated with better outcomes, regardless of whether patients received placebo or active interventions [78,79]. Within this review, it is unclear whether adherent patients generally do better, regardless of condition. It has been suggested that the mechanisms underlying a generalized adherence effect may be similar to the mechanisms that underlie the placebo effect [80], whereby the positive outcomes achieved from taking a placebo are the result of internalized beliefs about therapy, such as the expectations that the individual holds or the belief that the therapy will be effective. The placebo effect may also indicate a phenomenon of regression to the mean, in that outlying behavior returns closer to the mean over time. Alternatively, it has been suggested that adherence may be a more general indicator of orientation to healthy behaviors. Therefore, individuals who are adherent may be more likely to follow healthy lifestyle practices and therefore have improved outcomes [81,82]. Further exploration within the area of e-therapy needs to determine whether adherence influences outcomes through expectation beliefs or through participants being generally adherent to treatment recommendations. To do this, control group adherence behaviors need to be reported more.

### Limitations

We recognize that several key studies that were included in the original sample of 8300 were excluded as a result of selection criteria. Specifically, some trials that potentially would have met the review criteria were excluded before analysis as a result of database coding and indexing. While Cochrane-style systematic reviews have used rigorous data selection and extraction templates to select trials, the varied nature of the design and high attrition rate of e-therapies made it difficult to use such templates. Given this, the limits within the databases were used to ensure that the methodology of the review was as systematic and replicable as possible.

As the studies contained within this review were heterogeneous in terms of the populations studied, nature of the interventions used, lengths of follow-up, and outcome measures, we summarized the findings by adherence measure only, rather than attempting a meta-analysis. This has limited the ability to combine and report the data beyond the format used. Similarly, the large number of small studies included in the review may have been underpowered for finding significant results.

### Implications for Future Work

There are many difficulties in determining the role of adherence on outcomes in e-therapies. While objective data can be captured with relative ease, this may not truly reflect the user’s experience and dose. This is particularly pertinent when the user is required to complete activities such as homework exercises between online sessions, or when the user can print off material to review. In both of these scenarios, “objective measures” may underestimate the usage of the program. Similarly, user aspects such as processing speed, and familiarity with Web-based platforms and user interfaces are likely to influence the time spent online. Therefore, understanding the contributing factors of adherence is likely to be as important as understanding adherence per se.

From the findings of this review, it appears that the number of logins is the measure of adherence best correlated with physical outcomes, while module completion correlated with psychological outcomes. This suggests that program persistence and adherence may be important for physical and psychological interventions in different ways. However, these results need to be considered with caution, given the limitations of this review and the potential biases in the data. We therefore recommend that this be explored in future studies where adherence is the focus. It may mean that an aggregated adherence measure such as program exposure could provide a more meaningful measure of adherence by incorporating logins, time spent online, and number of activities and modules completed. However, it is not always clear how such a composite measure was derived in the studies and how each factor contributes to such a score. Similarly, variations existed within composite measure across each trial, making the comparison difficult. Furthermore, the design of interventions could take this into account by maximizing the likelihood of a participant undertaking the behavior most likely to enhance outcomes. For instance, maximizing module completion for depression and anxiety interventions, even at the expense of number of logins, may be a good trade-off. Understanding the differential effects of different measures of adherence will be important in future content and platform development, as well as in evaluating applicability and health service issues.

## Multimedia Appendix 1

Association of each type of adherence measure with outcome grouped by target of intervention
