# Supplementary material for: A Systematic Review of the Impact of Adherence on the Effectiveness of e-Therapies
Source: J Med Internet Res. 2011 Aug 5;13(3):e52. doi: 10.2196/jmir.1772 (PMC3222162; doi:10.2196/jmir.1772)
Supplement: Supplementary file 1 [file jmir_v13i3e52_app1.pdf]

Multimedia Appendix 1: Association of each type of adherence measure with outcome grouped by target of intervention

Target of Intervention: Fruit and Vegetable Consumption

Adherence Measure	*Study*	*N*	*Study Description*	*Outcome Measures*	*Measure of adherence used in analysis*	*Other measures of adherence collected or reported on but not analysed*	*Relationship between adherence and outcome*	*Strength of Adherence Measure*
Logins	Alexander, McClure et al. 2010 [32]	2,540	Population based three arm RCT with follow-up over 12 months. Intervention consisted of a website with information, interactive tools and videos. Arm 1 was the basic site, Arm 2 was a tailored version of the website with tailoring to needs and interest, and Arm 3 was the tailored website with motivational interviewing style e-mails supporting it.	Fruit and vegetable food frequency questionnaire Total fruit and vegetable consumption daily	Logins	Responses made to 17 program emails	Vegetable intake increased with more visits regardless of trial arm individual was allocated to – mean daily fruit and vegetable servings increased by 2.2 times for people that visited the website less than 6 times; by 2.5 servings for those that visited 7-13 times; and by 3.0 servings for those that visited the website 13 or more times.	+[b]

Target of Intervention: Physical Activity

Measure	*Study*	*N*	*Study Description*	*Outcome Measures*	*Measure of adherence used in analysis*	*Other measures of adherence collected or reported on but not analysed*	*Relationship between adherence and outcome*	*Strength of adherence measure*
Logins	Carr, Bartee et al. 2008 [33]; Carr, Bartee et al. 2009 [34]	67	RCT contained 4 arms – waitlist control, Traditional Exercise Program, ALED-I, or ALED-C. ALED programs consisted of a self-paced program with interactive activities and behaviour modification strategies lasting 16 weeks and drawing on motivational interviewing and social cognitive theory. ALED-I was delivered over the internet whilst ALED-C was delivered in the classroom. 16 week intervention with follow-up at 8 months.	Pedometer recording of physical activity<br>BMI<br>Waist circumference<br>X-ray absorptiometry<br>Metabolic measures of health<br>One mile walk test	Logins		At 8 months, number of logins was not associated with physical activity (p=0.13). Accessing the website on at least one occasion was positively correlated with physical activity at 8 months (P=0.02) and change in physical activity from baseline to 8 month follow-up (P=0.04).	+ [b]
	Marcus, Lewis et al. 2007 [36]; Lewis, Williams et al. 2008 [35]	249	Three armed RCT lasting 12 months. Participants allocated to tailored internet intervention, standard internet, or tailored print. Tailored intervention consisted of monthly prompts to return to site and get monthly feedback based on the transtheoretical model. Standard internet site contained links to other material, freely available to the public.	7 day physical activity recall<br>Balke protocol treadmill exercise test result	Logins	Time online per login, total time spent online, and activities completed.	Increased number of logins was correlated with increase in physical activity from baseline to 12 months, regardless of group (t=3.39, p<.01). Login was positively associated with an increase in median change in physical activity (β=34.32, CI=14.33-54.31) by log transformation.	
	McKay, King et al. 2001 [37]	78	RCT using diabetes patients. RCT consisted of two arms; the Diabetes	11-items related to work and non work activity from the Behavioural	Logins	Average weekly logins, average time spent	Using a dichotomy of 0-2 visits vs. 3 more visits, visiting the website on 3 or more occasions	

			Network Active Lives Intervention (D-Net) or internet information only control which included a glucose tracker. D-Net is an 8 week personalised program which leads participants through goal setting and activity scheduling. Participants also received 4 tailored messages over the course of the intervention. Participant forum also available.	Risk Surveillance System (BRFSS) used to calculate a total physical activity score<br>Centre for Epidemiological Studies – Depression Scale (CES-D)		online, number of posts sent to coach, number of posts sent to peers	lead to greater probability of increased physical activity. Usage was positively related to moderate/intense physical activity increase.	
Website Exposure	(McKay, King et al. 2001 [37]	78	RCT using diabetes patients. RCT consisted of two arms; the Diabetes Network Active Lives Intervention (D-Net) or internet information only control which included a glucose tracker. D-Net is an 8 week personalised program which leads participants through goal setting and activity scheduling. Participants also received 4 tailored messages over the course of the intervention. Participant forum also available.	11-items related to work and non work activity from the Behavioural Risk Surveillance System (BRFSS) used to calculate a total physical activity score<br>Centre for Epidemiological Studies – Depression Scale (CES-D)	Usage of program	Average weekly logins, average time spent online, number of posts sent to coach, number of posts sent to peers	Increased program usage related to moderate/intense physical activity increase.	+ [b]
Activities Completed	Carr, Bartee et al. 2008 [33]; Carr, Bartee et al. 2009 [34]	67	Two studies of RCT design. Study 1 contained 4 arms – waitlist control, Traditional Exercise Program, ALED-I, or ALED-C. ALED programs consisted of a self-paced program with interactive activities and behaviour modification strategies lasting 16 weeks and drawing on	Pedometer recording of physical activity<br>BMI<br>Waist circumference<br>X-ray absorptiometry<br>Metabolic measures of health<br>One mile walk test	Number of journal activities completed		At 8 months, journal activities completed ($p=0.06$) was not associated with physical activity at 8 months.	0 [c]

			motivational interviewing and social cognitive theory. ALED-I was delivered over the internet whilst ALED-C was delivered in the classroom. 16 week intervention with follow-up at 8 months.					

Target of Intervention: Nicotine Use

Adherence Measure	*Study*	*N*	*Study Description*	*Outcome Measures*	*Measure of adherence used in analysis*	*Other measures of adherence collected or reported on but not analysed*	*Relationship between adherence and outcome*	*Adherence measure strength*
Activities completed	Munoz, Barrera et al. 2009 [42]	1,000	4 arm RCT of smokers from the community. Arm 1 (Guia) – static website with cigarette counter, online journal and information about smoking and smoking cessation. Arm 2 – (Guia + ITEMs) consisted of GUIA plus individual emails with links to content. Arm 3 – (GUIA + ITEMS +MM) as per above with 8 lessons of cognitive-behavioural mood management. Arm 4 (GUIA+ITEMS+MM+VG) as per above with asynchronous bulletin board. Intervention ran for 12 months.	Self reported 7 day abstinence<br>Prolonged abstinence<br>Fagerström Test for Nicotine Dependence (FTND)<br>The MDE screener<br>The Centre for Epidemiological Studies Depression Scale (CES-D)	Activities completed		Abstinence at 1 months was associated with using cigarette counter 2+ times (Chi X = 6.99, P=0.0082), and with using journal (X=4, p=0.0456). Journal use was associated with abstinence at 3 months (X=3.99, p=0.485) and 6 months (X=10.49, p=0.0012).	+ [b]
Modules completed	Munoz, Barrera et al. 2009 [42]	1,000	4 arm RCT of smokers from the community. Arm 1 (Guia) – static website with cigarette counter, online journal and information about smoking and smoking cessation. Arm 2 – (Guia + ITEMs) consisted of GUIA plus individual emails with links to content. Arm 3 – (GUIA + ITEMS +MM) as per above with 8 lessons of cognitive-behavioural mood management. Arm 4 (GUIA+ITEMS+MM+VG) as per above with	Self reported 7 day abstinence<br>Prolonged abstinence<br>Fagerström Test for Nicotine Dependence (FTND)<br>The MDE screener<br>The Centre for Epidemiological Studies Depression Scale (CES-D)	Number of Modules completed (n=8)		Number of mood modules correlated was not associated with outcomes.	+ [b]

			asynchronous bulletin board. Intervention ran for 12 months.					
	Seidman, Westmaas et al. 2010 [47]	2,153	Two armed RCT looking at on online modular program of 32 sessions vs. a non-interactive site with downloadable information. Intervention program based on transtheoretical model and relapse prevention that provides individuals with a personalised quit plan. Intervention also contains interactive activities designed to enhance motivation.	7 day point prevalence of abstinence<br>30 day point prevalence prolonged abstinence<br>PANAS	Number of modules completed (n=32)		Controlling for covariates (such as depression) there was no difference in 30 day smoking prevalence in those that did not access the program and those that accessed the site 2-3 times. There was no difference in 30 day smoking prevalence between those that accessed the site 4-10 times or more than 10 times. Given this, analysis compared participants that accessed the site 0-3 times with those that accessed it 4 or more times. Logistical regression models for 30-day point prevalence indicated increased in the odds of quitting in participants that had accessed 4 or more modules when compared to those who had accessed up to 3 modules; at 4 month quit rates (OR 2.37, CI=1.59-3.52, p<0.001), at 7 m (OR = 2.11, CI=1.42-3.13, p<0.001), at 13m (OR = 1.66, CI=1.15-2.39, p <0.01). Similar results were found for prolonged abstinence	
	Strecher, McClure et al. 2008 [46]; Strecher, McClure et al. 2008 [77]	1,866	RCT of multiple arms based on components of a CBT and relapse prevention based program. Components varied on the level of tailoring the stories and content to individual participant characteristics. Follow-up was completed at 6 months.	Self reported 7 day point prevalence of abstinence	Number of sections opened (n=5)		Number of sections opened was correlated with smoking cessation at 12 months (OR= 2.26, CI 1.72-2.97). For each section opened there was an 18% increase in probability of quitting (OR 1.18, CI=.111-1.24). Heavy use of website (opened 3-5 sections) had a 37.4% cessation rate vs. light use with a cessation rate of	

							27.3%	
Website exposure	Severson, Gordon et al. 2008 [64]	2,523	RCT of two arm design. Intervention consisted of a website with an automated quit coach, videos, downloadable material, email prompts and patient forum. Control consisted of an information only website. Intervention lasted 6 months.	7 day point prevalence of tobacco use	Website exposure (number of logins, time spent online, number of forum posts, number of print requests)		Composite exposure (number visits and duration of visits) was strongly related to abstinence at 3 months (β=0.79, OR=2.20, CI=1.96-2.46, P<0.001) and 6 months (β=0.63, OR=1.88, CI=2.69-2.09, P<0.001). Enhanced exposure (number of visits, duration of visits, pages accessed, number of print pages accessed, number of postings) was correlated to abstinence at 3 (β=1.22, OR=3.40, CI=2.70-4.27, P<0.001) and 6 months (β=1.00, OR=2.72, CI=2.18-3.37, P<0.001) to a stronger degree.	+ [b]

Target of Intervention: Weight Management

Adherence Measure	*Study*	*N*	*Study Description*	*Outcome Measures*	*Measure of adherence used in analysis*	*Other measures of adherence collected or reported on but not analysed*	*Relationship between adherence and outcome*	*Adherence measure strength*
Activities completed	Tate, Wing et al. 2001 [39]	91	RCT of 2 arms. Participants were either assigned to internet education or an internet behaviour change program after initial face to face session. Internet behaviour change group also received weekly emails, online submission of activities and feedback via email. Follow-up occurred over 6 months.	Weight<br>Height<br>Waist Circumference<br>Paffenbarger Activity Questionnaire<br>The Centre of Epidemiologic Studies Depression Scale (CES-D)<br>Block Food Frequency Questionnaire	Activities completed		Total diaries submitted negatively correlated with weight at follow-up (r=-0.50, p=0.001).	++ [a]
	Webber, Tate et al. 2008 [40]	66	2 arm 16-week RCT. Arm 1 consisted of an online behaviour change program with weekly sessions to complete and bulletin board. Arm 2 contained the content of Arm 1 as well as weekly chat sessions.	Weight<br>Block food frequencies questionnaire (FFQ)<br>Paffenbarger Physical Activity Questionnaire<br>Marcus 5-item exercise self efficacy scale<br>Weight loss motivation scale of TSRQ<br>Online food and activity diaries	Activities completed (number of pages opened, number of posts made on forum)		Weight loss post intervention was associated diaries completed (r=0.64, p<0.01) and postings made to forum (r = 0.34, p=0.05).	
Logins	Tate, Wing et al. 2001 [39]	91	RCT of 2 arms. Participants were either assigned to internet education or an internet behaviour change program after initial face to face session. Internet behaviour change group also received weekly emails, online submission of activities and feedback via email. Follow-up occurred over 6 months.	Weight<br>Height<br>Waist Circumference<br>Paffenbarger Activity Questionnaire<br>The Centre of Epidemiologic Studies Depression Scale (CES-D)<br>Block Food Frequency Questionnaire	Logins		Login frequency significantly correlated with weight loss at 6 months in both the education (r=-0.33, p=0.03) and structured behaviour therapy group (r=-0.43, p=0.003).	++ [a]
	Tate,	92	RCT of 2 arm design.	Weight	Logins (Average		Logins were significantly	

	Jackvony et al. 2003 [38]		Participants were either assigned to a basic internet intervention or an internet behaviour change program with e-counselling via email. Standard internet group received tutorial on weight loss, tips and links each week and a directory of resources. Behaviour change group received the same intervention with additional e-mail counselling.	Height<br>Waist circumference<br>Venous blood glucose<br>Physical activity readiness questionnaire<br><br>Paffenbarger Activity Questionnaire<br>The Centre of Epidemiologic Studies Depression Scale (CES-D)	monthly logins)		correlated with weight change at 12 months for both groups; website + email group (r=-0.47, p <0.003); internet only group (r=-0.61, p<0.001).	
	Webber, Tate et al. 2008 [40]	66	2 arm 16-week RCT. Arm 1 consisted of an online behaviour change program with weekly sessions to complete and bulletin board. Arm 2 contained the content of Arm 1 as well as weekly chat sessions.	Weight<br>Block food frequencies questionnaire (FFQ)<br>Paffenbarger Physical Activity Questionnaire<br>Marcus 5-item exercise self efficacy scale<br>Weight loss motivation scale of TSRQ<br>Online food and activity diaries	Number of logins		Weight loss post intervention was associated with website visits (r=0.53, p<0.01).	

Target of Intervention: Depression

Adherence Measure	*Study*	*N*	*Study Description*	*Outcome Measures*	*Measure of adherence used in analysis*	*Other measures of adherence collected or reported on but not analysed*	*Relationship between adherence and outcome*	*Adherence measure strength*
Modules Completed	Andersson, Bergstrom et al. 2005 [45]	57	Two arm RCT. Arm 1 consisted of a 5-module, CBT intervention with each module ending with a quiz. Participants received email feedback on quiz. Each module must be completed before moving forward. Online asynchronous forum to discuss material. Arm 2 consisted of on online discussion group with different, unrelated content. Access to the next module was granted when the previous module was complete. Participants were followed up at 6 months.	Beck Depression Inventory (BDI)<br>Montgomery & Asberg Depression Rating Scale (MADRS-S)<br>Beck Anxiety Inventory (BAI)<br>Quality of Life Inventory (QoLI)	Number of modules completed (n=5)		Number of modules completed correlated weakly with post treatment BDI scores (Spearman's p=-0.33, P<0.05).	+ [a]
	de Graaf, Gerhards et al. 2009 [49]	303	RCT of 3 arms. Arm 1 consisted of 9 session computerised CBT program; Arm 2 was treatment as usual with GP, whilst Arm 3 was Computerised CBT plus treatment as usual by GP. Participants were advised to complete one session per week. Follow-up occurred over 6 months.	Beck Depression Inventory II (BDI-II)<br>Symptom Checklist 90 (SCL-90)<br>The Work and Social Adjustment Scale<br>Health Survey – Short Form (SF-36)<br>Dysfunctional Attitude Scale form A – 17 item version	Number of modules completed (n=9)	Logins, number of diaries completed, total time online, time online per login, number of other activities completed	Adherence was analysed with regards to BDI-II outcomes. In group by time interactions for dichotomous adherence variables (adherent vs. not adherent) was not significantly ($F_{6,148}$=0.85, P=0.53). When a less strict definition of adherence was used (participant completed 5 or more sessions of CCBT or 4 more with GP), there was still no significant interaction ($F_{6,242}$=1.67, P=0.13). Within group analyses with the relaxed definition of adherence as above, found an adherence x time interaction for the CCBT plus TAU group only	

							$(F_{3,88}=2.70, P=0.005)$. Contrast analysis found no difference on change scores for those that adhered and those that did not.	
	Meyer, Berger et al. 2009 [50]	396	Two armed RCT consisting of an intervention consisting of 12 modules of eclectic theoretical basis. Modules require constant user engagement in order to move forward. Control consisted of treatment as usual and delayed access. Follow-up occurred 12 months post intervention.	Beck Depression Inventory (BDI), German form without suicide item<br>Work and social adjustment scale, German version	Number of modules/sessions completed (n=8)		Number of sessions correlated with BDI change scores (r=0.91, p<0.001)	
	Warmerdam, van Straten et al. 2008 [75]	263	Two armed RCT. 1st arm consisted of a CBT program of 8 sessions completed over 8 weeks with a 9th session at week 12. 2nd arm consisted of problem solving therapy program of 5 sessions over 5 weeks. Follow-up completed 12 weeks after intervention is completed	The Centre for Epidemiological Studies Scale for Depression (CES-D)<br>The Hospital Anxiety and Depression Scale (HADS)<br>EuroQoL (EQ5D)	Number of modules completed (n=4)		No difference in depression scores at 12 week follow-up between those that completed all modules and those that did not	
	Spek, Nyklicek et al. 2007 [2007]; Spek, Cuijpers et al. 2008 [51]	301	Three armed, 10-week RCT consisting of internet based CBT, group therapy or waiting-list control. Internet CBT consisted of an 8 module program consisting of text, exercises, videos and images. Participants were able to complete the modules at their own pace.	BDI-II<br>WHO CIDI	Number of modules completed (n=12)		No program dose effect relationship found. The effect size for group therapy intervention improvement against waiting list group improvement was -0.07. Internet based intervention vs. waiting list was 0.52. Risk difference of 0.26 therefore 3.85 people have to undergo treatment to have a positive effect of 1. Group was 14.29	
	Christensen, Griffiths et al. 2006 [25]	2,794	Population based 6 armed RCT of an internet based CBT program based on MoodGym. MoodGym	Goldberg Depression Scale<br>Goldberg Anxiety Scale	Modules completed		Linear regression analysis of anxiety/depression scores as outcomes and number of modules completed as	

			consisted of five modules, with a combination of these modules comprising the 6 arms of the RCT. Program consists of activities and psychoeducation.				predictor. Completion of modules predicted final scores up to 4 modules. No difference between 4-5 modules on outcomes	
	van Straten, Cuijpers et al. 2008 [57]	213	Two armed RCT consisting of an active intervention with a delayed access waitlist control. Intervention consisted of a 4 week problem solving therapy program with email feedback.	The Centre for Epidemiologic Studies Depression Scale (CES-D)<br>Major Depression Invention (MDI)<br>Symptom Checklist anxiety subscale (SCL-A)<br>HADS – anxiety questions only<br>Maslach Burnout Inventory (MBI) – Dutch version<br>European Quality of Life Questionnaire (EQ5D)	Completion rate of modules		Effect size (outcome measures) was largest for those that completed the intervention. Effect sizes, post intervention; CES-D(*d*=0.67); MDI (*d*=0.56).	
Website exposure	de Graaf, Huibers et al. 2009 [65]	200	three armed RCT utilising the Colour Your Life CBT program which consists of an interactive combination of text, audio and video over 8 weekly sessions with a 9th booster session, and 13 homework assignments. Arm 1 consisted of unsupported CBT, Arm2 consisted of treatment as usual and CBT and Arm 3 was treatment as usual. Intervention lasted 12 weeks with follow-up at 9 months	BDI-II<br>Credibility/Expectancy Questionnaire (CEQ)<br>Expectancy Questionnaire	Number of modules completed (n=9), time spent online, number of homework activities completed, logins, diaries completed, time spent online per login.	Time spent online, number of homework activities completed, logins, diaries completed, time spent online per login.	Adherence was correlated with depression outcomes at 3 and 9 months using a stepwise logistic regression. At 3 months, it was found that more than 12 logins (OR=2.77, CI=1.44-5.32, P=0<0.01), greater than 173 minutes total time spent online (OR=2.15, CI=1.12-4.14, P=<0.05) and completion of more than 26 mood diaries (OR=2.74, CI=1.42-5.29, P=0<0.01) was correlated with depressive improvement. At 9 months, improvement with depressive symptomology was associated with more than 3 sessions completed (OR=2.30, CI=1.19-4.46, P=0<0.05), completion of more than 4 homework activities (OR=2.49, CI=1.28-4.84, P=0<0.01), more than 12	+ [b]

							logins (OR=2.18, CI=1.14-4.18, P=0<0.05), greater than 173 minutes total time spent online (OR=2.25, CI=1.14-4.44, P=<0.05) and completion of more than 26 mood diaries (OR=1.96, CI=1.02-3.77, P=0<0.05)	
Activities completed	Andersson, Bergstrom et al. 2005 [45]	57	Two armed RCT. Intervention consisted on 5 module CBT depression with emailed feedback on homework exercises. Participants were unable to access the next module for 24 hours after completing the previous module. Control consisted of an online discussion group covering general health topics. Follow-up over 6 months.	Beck Depression Inventory (BDI)<br>Montgomery & Asberg Depression Rating Scale (MADRS-S)<br>Beck Anxiety Inventory (BAI)<br>Quality of Life Inventory (QoLI)	Forum contributions		Online activity in discussion group was not correlated to outcomes.	0 [c]
Logins	Clarke, Reid et al. 2002 [41]	299	Cognitive therapy RCT consisting of two arms. Intervention was a self paced skills training program with 7 chapters based on cognitive restructuring. Program provided user with ability to store information, provided feedback over time and to view cartoons of the basics of cognitive restructuring. Control was treatment as usual with like to website to obtain general information. Participants followed-up over 32 weeks.	The Centre for Epidemiologic Studies Depression Scale (CES-D)	Logins	Mean and median number of modules completed (n=7)	A random effects regression analysis found no significant dose effect (F=0.95, P=0.33 for sign-in-by-time).	0 [c]
	Clarke, Eubanks et al. 2005 [23]	255	Cognitive therapy RCT consisting of three arms. Intervention 1 was a self paced skills training	The Centre for Epidemiologic Studies Depression Scale (CES-D)<br>Short Form 12 (SF-12)	Logins	Mean and median number of modules completed (n=7)	No significant relationship was found between logins and CES-D or SF-12 at any time point.	

			program with 7 chapters based on cognitive restructuring. Program provided user with ability to store information, provided feedback over time and to view cartoons of the basics of cognitive restructuring. Participants were sent postcard reminders to login. Intervention 2 was as above with telephone follow-up instead of postcards. Control was waitlist. Participants followed-up over 16 weeks.					
Time online	Clarke, Kelleher et al. 2009 [58]	160	Two armed RCT with intervention and treatment as usual control. The intervention was a self guided behavioural and cognitive therapy tutorials over four modules containing psychoeducation, journal section and interactive activities. Follow-up occurred over 32 weeks.	PHQ-8	Time spent online	Mean and median number of sessions completed	Reduction of symptoms as reported by the (PHQ-8) was associated with less time online ($F_{1,221}$=5.84, P=0.02).	- [d]
Pages opened	Clarke, Kelleher et al. 2009 [58]	160	Two armed RCT with intervention and treatment as usual control. The intervention was a self guided behavioural and cognitive therapy tutorials over four modules containing psychoeducation, journal section and interactive activities. Follow-up occurred over 32 weeks.	PHQ-8	Number of pages opened	Mean and median number of sessions completed	Reduction of symptoms as reported by the (PHQ-8) was associated with fewer page hits ($F_{1,221}$=8.35, P=0.004).	- [d]

Target of Intervention: Infertility Associate Distress

Adherence Measure	*Study*	*N*	*Study Description*	*Outcome Measures*	*Measure of adherence used in analysis*	*Other measures of adherence collected or reported on but not analysed*	*Relationship between adherence and outcome*	*Adherence measure strength*
Time online	Cousineau, Green et al. 2008 [60]	190	Solomon 4 design RCT consisting of a psychoeducation website and a delayed access waitlist control. Participants were asked to access the site for two 45 minute periods over a 4 week period.	Fertility Problems Inventory (FPI)<br>Infertility Self Efficacy Scale (ISE)<br>State Trait Anxiety Inventory (STAI)<br>Ways of Coping Scale (WOC)<br>Revised Dyadic Adjustment Scale (RDAS) Dyadic Cohesion Subscale<br>Perceived Negative Support Scale (PNSS)<br>Decisional Conflict Scale	Time spent online	Number of logins	Participants dichotomised into those that spent more than 60 or more minutes on the website and those that spent less than 60 minutes on the website. Those that spent 60 or more minutes on the website reported lower global stress scores on the FPI (t_{44}=2.27, P=0.028, d=0.68), lower FPI rejection of childfree lifestyle scores (t_{44}=2.33, P=0.025, d=0.070), and greater gains on the ISE (t_{44}=-2.34, P=0.024, d=0.71),	+ [b]

Target of Intervention: Anxiety Disorders

Adherence Measure	*Study*	*N*	*Study Description*	*Outcome Measures*	*Measure of adherence used in analysis*	*Other measures of adherence collected or reported on but not analysed*	*Relationship between adherence and outcome*	*Adherence measure strength*
Modules completed	van Straten, Cuijpers et al. 2008 [57]	213		The Centre for Epidemiologic Studies Depression Scale (CES-D)<br>Major Depression Invention (MDI)<br>**Symptom Checklist anxiety subscale (SCL-A)**<br>**HADS – anxiety questions only**<br>Maslach Burnout Inventory (MBI) – Dutch version<br>European Quality of Life Questionnaire (EQ5D)	Completion rate of modules		Effect size (outcome measures) was largest for those that completed the intervention. Effect sizes, post intervention; SCL-A (*d*=0.51); HADS (*d*=0.48) and EQ5D (*d*=0.44).	+ [b]
	Carlbring, Nilsson-Ihrfelt et al. 2005 [55]	49	Two armed RCT of an online CBT intervention vs. face-to-face therapy. Online intervention	Agoraphobic Cognitions Questionnaire (ACQ)<br>Body Sensations Questionnaire (BSQ)	Number of modules completed (n=10)		There was no correlation between number of modules completed an any of the change scores on outcome measures (all r's<0.26,	

			consisted of 10 modules with essay exercises, quizzes at the end of each module, posting on an online discussion and email feedback. Face-to-face arm consisted of 10 weekly individual sessions with homework exercises and playbacks of session recordings. Follow-up occurred one year post treatment/	Mobility Inventory (MI)<br>Beck Anxiety Inventory (BAI)<br>Beck Depression Inventory (BDI)<br>Quality of Life Inventory (QoLI)<br>Clinical Interview (SCID)			all p's>0.21)	
	Furmark, Carlbring et al. 2009 [56]	235	RCT consisting of several arms. Arm 1 was an internet based CBT program consisting of 9 consecutive modules. Each module consisted of information, a homework exercise and a quiz. Participants also submitted a summary of the module via email. Participants also had access to an online forum. Arm 2 consisted of pure bibliotherapy where participants received the self help manual by mail. Follow-up occurred over a year. Arm 3 consisted of a waitlist control. Arm 4 was bibliotherpay with access to an online discussion group. Arm 5 was internet delivered applied relaxation consisting of 9 modules. Follow-up occurred over a year.	Liebowitz Social Anxiety Scale – Self Report (LSAS-SR)<br>Social Phobia Scale (SPS<br>Social Interaction Anxiety Scale (SIAS)<br>Social Phobia Screening Questionnaire (SPSQ)<br>Beck Anxiety Inventory (BAI)<br>Montgomery & Asberg Depression Rating Scale (MADRS-S)<br>Quality of Life Inventory (QOLI)	Number of modules completed (n=9)		Modest correlations were found between the number of completed modules and change scores for anxiety measures (assumed to be all measures) at post intervention and at follow-up.<br>Correlations were still significant at post-intervention (r=0.15-0.29, P=0.07-0.002, *n*=154) and at the one year follow-up (r=0.23-0.29, P=0.004-7-0.002, *n*=154)	
	Tillfors, Carlbring et al. 2008 [52]	38	Two armed RCT comparing online CBT with and without exposure. Online program consisted of 9 modules which consisted of information, exercises and	Liebowitz Social Anxiety Scale Self Report Version (LSAS-R)<br>Social Phobia Scale (SPS)<br>Social Interaction Anxiety Scale (SIAS)	Number of modules completed (n=9)		Pearson's correlations were used to analyse the relationship between number of modules completed and outcomes at both post-intervention and follow-up. No significant result was found	

			essay questions. Participants were also asked to summarise their weekly learnings and post on a forum. Feedback on homework assignments was emailed. Exposure group had five live group exposure sessions. Follow-up occurred over one year.	Social Phobia Symptoms Questionnaire (SPSQ) Beck Anxiety Inventory (BAI) Quality of Life Inventory (QoLI)			for the group without face-to-face contact.	
	Titov, Andrews et al. 2008 [54]	88	Two armed RCT of online CBT vs. waitlist control. 10 week program of six online modules with homework assignments, participation in an online discussion and email feedback from therapist. Each session provided a story about a character with social phobia, information, homework and a printable summary. Responses made by email unless indicated otherwise.	Social Interaction Anxiety Questionnaire (SIAS) Social Phobia Scale (SPS) Kessler-10 (K-10) Patient Health Questionnaire (PHQ-9) World Health Organisation Disability Assessment Schedule II (WHODAS-II) Anxiety Change Expectancy Scale (ACES) Credibility/Expectancy Change Questionnaire (CEQ)	Number of modules completed (n=6)		Completing the program did not appear to have a difference on change scores (t_{89}=-0.24-1.46, p<0.16-0.85).	
	Titov, Andrews et al. 2008 [55]	98	Three armed RCT of online CBT with or without clinician assistance, vs. waitlist control. 10 week program of six online modules with homework assignments, participation in an online discussion and email feedback from therapist. Each session provided a story about a character with social phobia, information, homework and a printable summary. Responses made by email unless indicated otherwise. The clinician assisted arm contained complete program as described above. Those	Social Interaction Anxiety Questionnaire (SIAS) Social Phobia Scale (SPS) Kessler-10 (K-10) Patient Health Questionnaire (PHQ-9) Sheehan Disability Scale (SDS) Anxiety Change Expectancy Scale (ACES) Credibility/Expectancy Change Questionnaire (CEQ)	Number of modules completed/lessons /sessions (n=6)		Dichotomised participants into completers and non-completers. Completers had significantly better outcomes at post intervention on self rated avoidance (t_{28}=2.96,p<0.001), SPS (t_{28}=3.09,p<0.001), and SDS (t_{28}=2.12,p<0.05). Pearson's correlation coefficients were calculated to determine the relationship between number of sessions completed and change scores on outcome measures. Significant relationships were found between the number of lessons and changes in SPS (r=0.47, p<0.008); lessons and events avoided (r=0.51, p<0.004) and lessons and events attended (r=0.52, p<0.003).	

			without assistance did not receive feedback or support from therapist.					
Time online	Kenardy, McCafferty et al. 2006 [59]	83	Three armed RCT consisting of Panic Online (6 week online CBT program for panic disorder) with email follow-up, a manualised CBT workbook with telephone follow-up, and an information only control. Follow-up occurred at 3 months.	Anxiety Sensitivity Index (ASI)<br>Bodily Sensations Questionnaire (BSQ)<br>The Catastrophic Cognitions Questionnaire-Modified (CCQ)<br>The Agoraphobic Cognitions Questionnaire (ACQ)<br>The Centre for Epidemiologic Studies Depression Scale (CES-D)	Time online	Number of modules completed (n=6)	No relationship was found between time spent online and outcome measures.	0 [c]

Target of Intervention: Body Dissatisfaction/Eating Disorder Risk

Adherence Measure	*Study*	*N*	*Study Design*	*Outcome Measures*	*Measure of adherence used in analysis*	*Other measures of adherence collected or reported on but not analysed*	*Relationship between adherence and outcome*	*Adherence measure strength*
Time online	Low, Charanasomboon et al. 2006 [61]	72	RCT examining the Student Bodies (SB) program. SB is an 8 weekly module plus one booster session. The program is interactive, tailored and based on CBT. IT utilises text, audio, online journals, self assessment and behavioural assignments. Arm 1 consisted of SB with an additional moderated discussion group. Arm 2 was SB with an unmoderated discussion group, and Arm 3 was SB without a discussion group. Follow-up occurred at 8-months.	Eating Disorders Inventory Drive for Thinness Subscale (EDI-DT)<br>Eating Disorders Inventory Bulimia Subscale (EDI-B)<br>Eating Disorders Inventory Body Dissatisfaction Subscale (EDI-BD)<br>The Weight and Shape Concerns Scale (WSC)<br>Stunkard Figure Rating Scale<br>Sociocultural Attitudes Towards Appearance Scale (SATAQ) Internalization and Awareness Subscales<br>Body Mass Index (BMI)	Time online.		Time online was significantly correlated with decrease in EDI-DT ($r=0.25$, $p<0.05$) and lower reported weight and shape concerns at long term follow-up ($r=0.27$, $p<0.05$). No association with time online with change in EDI-B or internalisation of thinness.	+ [b]
Pages opened	Taylor, Bryson et al.	480	RCT consisting of a 9 module CBT program vs. a	The Weight and Shape Concerns Scale (WSC)	Pages read	Attendance at booster session.	Adherence was correlated with reduced post-intervention scores	+ [b]

	2006 [62]		waitlist control. Intervention consisted of SB (as outlined above with discussion group). Follow-up occurred over 3-years.	EDE Questionnaire (EDE-Q)<br>Eating Disorders Inventory Drive for Thinness Subscale (EDI-DT)<br>Eating Disorders Inventory Bulimia Subscale (EDI-B)<br>The Centre for Epidemiological Studies Depression Scale (CES-D)<br>Multidimensional Scale of Perceived Social Support<br>BMI			on WCS (t_{209}=-2.3, p=0.02), EDE-Q restraint subscale (t_{211}=-3.0, p=0.003), and EDE-Q weight concerns subscale (t_{211}=-2.3, p=0.02).	
	Zabinski, Wilfley et al. 2004 [63]	56	Two armed RCT of active intervention and delayed access control Intervention consisted of Student Bodies as described previously. Follow-up occurred over 10 weeks.	Body Shape Questionnaire (BSQ)<br>Eating Disorders Inventory Drive for Thinness Subscale (EDI-DT)<br>Eating Disorders Inventory Bulimia Subscale (EDI-B)<br>Eating Disorders Examination Questionnaire (EDEQ) weight concerns subscale<br>Eating Disorders Examination Questionnaire (EDEQ) shape concerns subscale<br>Eating Disorders Examination Questionnaire (EDEQ) restraint subscale<br>Eating Disorders Examination Questionnaire (EDEQ) eating subscale<br>BMI	Percentage of web pages opened.		No significant relationship found between measures of adherence and outcome measures.	
Activities Completed	Winzelberg, Eppstein et al. 2000 [44]	60	Two armed RCT of active intervention and waitlist control Intervention consisted of Student Bodies as described previously. Follow-up occurred over 3 months.	**Body Shape Questionnaire (BSQ)**<br>Eating Disorders Inventory Drive for Thinness Subscale (EDI-DT)<br>Eating Disorders Inventory Bulimia Subscale (EDI-B)<br>Eating Disorders Examination Questionnaire (EDEQ) weight concerns	Percentage of activities completed		Stepwise regression analysis showed that treatment adherence accounted for 14% variance of BSQ at follow-up (*β*=-0.299), (F(2,18)=5.71, p=0.03)	+ [b]

				subscale<br>Eating Disorders Examination Questionnaire (EDEQ) shape concerns subscale<br>BMI				
	Zabinski, Wilfley et al. 2004 [63]	56	Two armed RCT of active intervention and delayed access control Intervention consisted of Student Bodies as described previously. Follow-up occurred over 10 weeks.	Body Shape Questionnaire (BSQ)<br>Eating Disorders Inventory Drive for Thinness Subscale (EDI-DT)<br>Eating Disorders Inventory Bulimia Subscale (EDI-B)<br>Eating Disorders Examination Questionnaire (EDEQ) weight concerns subscale<br>Eating Disorders Examination Questionnaire (EDEQ) shape concerns subscale<br>Eating Disorders Examination Questionnaire (EDEQ) restraint subscale<br>Eating Disorders Examination Questionnaire (EDEQ) eating subscale<br>BMI	Percentage of required posts completed.		No significant relationship found between measures of adherence and outcome measures.	

[a] at least two studies finding a positive correlation between increased adherence and outcome measures;
[b] one study or mixed evidence with predominantly positive relationships found between adherence measures and outcome;
[c] no relationship found between adherence and outcome measures;
[d] one study or mixed evidence with predominantly negative relationships found between adherence measures and outcome;
[e] two studies finding a negative correlation between increased adherence and outcome measures.
